# Collectanea Medica, Consisting of Anecdotes, Facts, Extracts, Illustrations, Queries, Suggestions, &c.

**Published:** 1817-01

**Authors:** 


### COLLECTANEA MEDICA,

CONSISTING OF

### ANECDOTES, FACTS, EXTRACTS, ILLUSTRATIONS, QUERIES, SUGGESTIONS, &c.

RELATING TO THE

*History or the Art of Medicine, and the Auxiliary Sciences.*

Quicquid agunt medici, nostri farrago libelli.

WE have so much continental matter, and some of it has been so long on our shelf, that we must dedicate to it and our remarks the division of Collectanea for this month. We have selected such as are immediately or remotely connected with contagion.

The first is a communication from Dr. Von Embden, of Hamburgh, containing "*Observations on the Nature and Treatment of Syphilis;* by Dr. ALLINHOFER, Physician to a Lock at St. Petersburgh.

"Chancre poison, given to dogs and cats, internally, mixed with bread and milk, produced no disturbance in their state of health; but it killed greenfinches and sparrows. A budding rose, when touched with it, faded immediately. Leeches did not suck blood that was mixed with it, and dropped off instantly when touched with it. Milk, warm from the cow, was by it brought to curdle directly. With milk just drawn from the vein it mixed so intimately, that it could not be distinguished, and promoted its coagulation. Oxymuriatic acid, nitrate of quicksilver, and a saturated solution of oxymuriate of quicksilver, destroyed it immediately. When mixed with tepid water, it was precipitated after a little while. Gonorrhœal matter first floated on the water, but, when stirred, it united with it, forming a turbulent whitish flaky liquid. Inoculation of chancre poison in a dog, produced upon a red ground a small pimple filled with pus, but more like a variolous pustule than a chancre, and soon healed again. A wound in the leg of a dog got an unhealthy appearance, through the introduction of syphilitic poison into it; several phagedenic ulcers arose around it, but had more the appearance of scrofula than of syphilis; the dog died, in a week after, with atrophic symptoms. Gonorrhœal matter, introduced into the urethra of a dog, produced a similar discharge, which lasted a few days; introduced into a wound, it appeared to accelerate the cure. Syphilis seems to be nearer related to scurvy than to scrofula;—thus do chancres, in scorbutic subjects, always assume the appearance of that morbid form. Its deap seat, pale circumference, dark red edges, an d perpetual tendency tendency to suppurate, plainly mark a chancre; whereas the corrosion of the ulcer, the fluidity of the pus, the tendency to bleed, and the titillating pain, signalise its scorbutic complication. Matter, from a scorbutic ulcer, applied to a simple chancre, renders it unhealthy, and produces such a change in it, that mercurials cannot be made use of for some time. Phagadenic water, mixed with scrofulous matter, leaves the simplest chancres uncured, but makes them rather more unclean; but syphilis maintains its own form in scrofulous patients, and produces peculiar ulcers next to the scrofulous ones; neither does scrofulous matter produce any alteration in syphilitic ulcers. In dying people, Dr. A. always observes the chancres to become dry, the pale red ring to disappear, and buboes and swelled testicles, if ever so hard, to become soft, provided they were not scirrhous. The latter symptom he considers as a certain prognostication of approaching death.

"In the bodies of those who had both chancres and buboes, he found, after death, the absorbents, from the genitalia to the inguinal glands, not only inflamed, but as it were corroded, the lymph thus appearing to be more than a mere conductor of the syphilitic poison.

"Fresh vaccine matter, applied to a simple recent chancre, caused a burning, which went off again after half a minute, followed by great itching, which lasted for half an hour; whereupon the chancre directly appeared already changed into a healthy ulcer, and, after three days, no further mark than a scar was perceptible, though no remedy whatsoever had been applied. In chancres of long standing, and such as had already been treated with antisyphilitics, vaccine matter had no effect whatsoever. Variolous matter produced no alteration. The progress of local syphilis, at other times so rapid, was at a stand during vaccination.

"What regards the treatment, Dr. A. makes use only of mercurial preparations, having been taught, by experience, that all other remedies recommended against this disorder do not answer the purpose. Among 1000 cases, he met only with four, where, from a peculiar idiosyncrasy against quicksilver, the application of acids became necessary; and with eight, where mercurials, having produced ptyalism as early as the fourth or sixth day, acids were obliged to be resorted to.

"No advantage was derived from decoctions of barks and roots, except a concentrated decoction of the cort. salicis, in syphilitic gout and exostosis. It indeed dissolves the tophi, and relieves nocturnal head-aches, even without the use of mercury; yet they return again after a while. It does effect a radical cure, when given in conjunction with the mineral, particularly the hydrargyrum nitricum. This latter preparation operated almost specifically in obstinate syphilitic rheumatalgia. Of 100 patients who had already laboured many years under this painful disorder, 63 were perfectly cured; and the cure, which seldom required longer than a month, was perfect and permanent. He dissolves one part of the the metal in two of nitric acid, of which he gives from one to three drops, night and morning. A morbid state of the organs of respiration or digestion, and general debility of the organism, prohibit the use of this preparation. Sometimes, though but seldom, it produces vomiting, diarrhœa, and colic, which are to be obviated by opiates. Swelling of the gums, and ptyalism, generally take place about the tenth, seldomer as soon as the seventh, day;—the remedy is then to be laid aside, and resumed when these symptoms have disappeared.

"The great utility of gonorrhœal inoculation, in cases of metastasis, Dr. A. found confirmed by manifold experience. Among others, he relates an instance of a man, who, the gonorrhœa having been stopped after catching cold, suddenly lost the hearing of the left ear, which, after a few days, filled with a fluid like gonorrhœal matter. This same fluid was inoculated in the urethra, and, after twelve hours, the former discharge re-appeared, with great scaldings; and, on the following day, the running from the ear disappeared, and hearing was restored. In gonorrhœa of long standing, and even in simple ones, he recommends injections of a solution of lapis divinus in goulard-water as particularly useful; besides which, in very obstinate cases, a small blister is to be applied to the perinæum. Indurated buboes are to be softened and maturated by cataplasms of black bread and vinegar, or, which is still more effective, by sinapisms.

"In gangrenous buboes, the following method is said to be very efficacious:—powdered calmus is to be strewed in the wound, in one or more lines thickness, which is to be moistened with oil of turpentine, until it becomes like a poultice. When the edges of the wound become much inflamed and projecting, they are to be covered with cerate of lead, which mode of treatment is to be persevered in till every vestige of gangrene and impure suppuration is gone; after which the wound is to be quickly healed up with goulard water."

*** The above is worthy of recording, but most of our readers will see the deficiency of the paper, inasmuch as the local appearances of none of the diseases mentioned are sufficiently marked to ascertain their true character.

The next paper shows that, if we often accuse our countrymen of confused terms and want of arrangement, they are complete philologists and logicians, compared with the French pathologists.

"*Is the Yellow Fever contagious?* The history of yellow fever involves an important question, which it would be most desirable to solve, viz. whether or not it is contagious? This solution, so interesting to all nations, is particularly to be sought after by those whose commercial communications are frequent with those countries where the fever is prevalent. Physicians of eminence, such as Chisholm, Wright, Ling, Currie, Makittrick, Pugnet, Aregula, Palloni, Cailliot, Thebaut, Bally, &c. maintain its contagious tagious nature; while others, of not less repute, as Devèze, Valentin, Miller, Dalmas, Smith, Savaresi, &c. entertained a contrary opinion. Again, Gilbert, Clark, Humboldt, and many others, have thought that the fever in question is only *contagious* under certain conditions of locality and temperature. Rush is well known to have first adopted, and afterwards abandoned, the contagious creed. M. Moreau de St. Méry asserts, however, that Dr. Rush declared, on his death-bed, that he had denied contagion for a particular reason, although he had never, for a moment, altered his belief in the infectious nature of yellow fever. He disavowed, in his last moments, every thing which he had written in favour of non-contagion. 'Il a désavoué a son heure supreme, tout ce qu'il avoit ecrit en faveur de la non contagion.'

"All these authors had opportunities of seeing the disease, both in America and Europe, and speak from experience. We shall now give a fair exposition of the arguments on both sides. We believe, however, that much of the discrepancy of opinion among physicians on this point has arisen from the different ideas attached to the word contagion. Thus some pathologists admit, that the matter of contagion may be transmitted to a certain distance, through the medium of the air, while the greater number consider it as resulting only from mediate or immediate contact—contagium a contactu. Are there not many diseases, for instance the plague and typhus, which may be communicated in either way?

"According to Ulloa (Relacion Historica del Viage a la America Meridional, &c.), the South-Sea galleons, having sailed from Panama, in 1740, for the purpose of placing the treasure in security at Guayaquil, carried to this latter colony the yellow fever, which there committed great devastation, especially among the seamen and strangers, the people of the country being nearly exempt from its influence. In 1741, some strangers introduced the disease into Malaga, by means of merchandize. (Villalba, Epidemologia.) Lind reports, that the body of a young man who died at Barbadoes, of yellow fever, having been opened at Philadelphia, spread the disease throughout the city. The same physician speaks of that which prevailed at Cadiz in 1764, as having been imported from America. The fever which broke out in the United States in 1793, was, according to Bally, introduced there by the French colonists, who emigrated thither from the West Indies, to escape massacre. The corvette, Dolphin, which conveyed the governor of Havannah to Cadiz, carried to this last place the fever under consideration, which first attacked those who had communication with the baggage, but afterwards spread through the city. Great numbers of the inhabitants, who fled in all directions into the adjacent villages and country, carried the contagion along with them. Whole families, who remained in Cadiz, but kept themselves insulated, escaped the fever. (Aregula.) During the epidemic at Leghorn in 1804, we are assured, that those who avoided all communication with the sick were unaffected by by the contagion. (Palloni.) In 1802, a new epidemic ravaged Philadelphia, shortly after the arrival of a packet vessel from Cape François. (Bally.) Aregula observed at Malaga, 1803, that, on every Monday, a great number of people fell ill, from their meeting together in the churches the preceding Sunday. The yellow fever prevailed at Antequerra in 1804. After a solemn procession for imploring divine interposition, the mortality was more than doubled.

" The learned M. Thiebaut de Berneaud, who was present at Leghorn in 1804, when yellow fever desolated that city, and who addressed, on this occasion, a most animated epistle to Professor Desgenettes, entertains no doubt of the disease having been imported. We shall here give some important details of M. Thiebaut, relative to this importation.

" On the 18th of August, 1804, the Spanish ship Anna Maria cast anchor in the Port of Leghorn, on her return from the Havannah. During the voyage, almost the whole crew died of yellow fever. Having previously touched at Cadiz, she was refused entrance there; but, what was still more inhuman, she was furnished with a fresh crew, and a bill of health! A few days after casting anchor in Leghorn roads, two sick men quitted the vessel, and went to lodge at an inn. Three days after their debarkation they died. Twelve others, who lodged in the same house, fell in succession; and the inn itself became a focus of contagion. A Neapolitan, who lodged at this inn, decamped, in order to escape the disease; but, six days after his departure, he was seized with yellow fever, and died. A baker, of Leghorn, having sold some biscuit on-board the Spanish ship, left there the bags in which it was contained, during two days, when they were brought on shore, and slept upon by the workmen of the bakehouse. These all died, as did the baker and his wife, the infection spreading through the whole house.

" M. Pachaud, of Nice, a rich merchant, settled at Leghorn, purchased, from his peruke-maker, a plume of feathers that had been imported in the Anna-Maria from America. He was seized with the yellow fever, and died, as did his wife and servant. The perukier shared the same fate.

" A French butcher, who lodged at the inn already mentioned, died of the yellow fever, as did his wife. A French officer, and the mistress of the inn, who had visited the two persons abovementioned, during their illness, survived them only four days.

" The officers of health that were stationed on board the ship during her twelve days' quarantine, almost all the caulkers that had been employed in caulking the vessel, and several inhabitants of the houses on the Mole, were seized with yellow fever and died.

" The ship was laden with sugar, logwood, sarsaparilla, hides, &c. which goods were landed and deposited in two stores, situated in different streets. The fever committed uncommon ravages in these these two streets. Two porters employed in transporting these goods died, between the fourth and seventh days; and the man who had charge of the stores died on the second day. Such are the principal facts on which M. Thiebaut grounds his proofs of the yellow fever of Leghorn being contagious.

"M. Moreau de St. Méry, who lived in the midst of the most destructive epidemics of the yellow fever, for thirty years, at Martinique, St. Domingo, and Philadelphia, has often seen proofs of the yellow fever being contagious; but adds, that this dreadful character does not appertain to every epidemic of that disease.

"M. Moreau de Jonnes, whose testimony also is entitled to the greatest respect, is convinced that this fever is sometimes contagious; but he has seen epidemics without any infectious properties.

"In the memorable epidemics of 1802 and 1803, which ravaged the West Indies and several parts of the United States, the yellow fever, he maintains, was certainly contagious. Of thirty-two persons attached to the etat-major of the army in Martinique, thirty-one died, leaving M. Moreau the only survivor. At this period, the medical officers were almost all carried off by the fever. The following authentic fact, communicated to us by M. Moreau de Jonnes himself, seems very conclusive on the question of contagion. The French brig Palinurus having, in the year 1808, moored in the harbour of Fort Royal, Martinique, was soon visited by the yellow fever. The mortality being considerable, the governor ordered her out on a cruize, in hopes of checking the disease. While at sea, she fell in with the English brig the Carnation, coming from Europe, and an engagement ensued. The French captain boarded, and captured the Englishman; and the greater part of the crew of the latter were, of course, transferred to the former. Of these, a great number were seized with yellow fever. In this case, no doubt could be entertained of the disease having been communicated by contagion,—since the English sailors had not set foot in any part of the West Indies, and, consequently, had not been exposed to the causes of yellow fever, except on board the Palinurus.

"We shall close the evidence on this side of the question, by adducing the opinion of M. Desgenettes, who has pushed his researches on the subject to the greatest length, especially as far as regards the yellow fever of Spain and Portugal. He founds his opinion of the double nature of the disease on the observations of competent judges, who have seen this fever originate and develope itself in the middle of the Peninsula, particularly in the fifteenth and sixteenth centuries, without the possibility of communication with other infected countries; and, on the other hand, on the demonstrative proofs of importation and contagion, which, of late years, have multiplied so as to remove all doubt from the mind of scepticism itself. He refers to the inestimable work of Dr. Joachim de Villalba, entitled Epidemiologia Espanola, Madrid, 1803, who traces back as far as the year 476, B. C.; also to the 11th vol. of Corvisart's Journal.

"We now come to the authorities for the non-contagious character of yellow-fever. M. Louis Valentin, well known by his excellent writings, and who has published a distinct treatise on yellow fever, denies contagion in every instance. This gentleman has communicated to us the following facts and reasons, on which he grounds his opinion.

"To resolve this question, it is necessary to examine it with a mind untainted by prejudice or partiality, and disciplined by long experience. It is by seeing yellow fever several times, in various places, and under all its different aspects, that we can, with any prospect of success, attempt to remove the veil of obscurity from this subject of contention among European physicians. As those who have studied and observed this fever most, are evidently entitled to the greatest degree of credit, so the medical practitioners of North and South America have a manifest advantage over all others.

"Among the physicians of the United States, there is scarcely a dissentient voice, excepting two or three of the elder members of the college of Philadelphia, particularly Drs. Currie and Hosack. Of Dr. Rush it is needless to speak. The Spanish physicians, Desessé and Mocino, who have resided a long time, the one in Mexico, the other in Peru, where they had abundant opportunity of observing yellow fever, assert, that it is entirely devoid of any contagious character. M. Humboldt also has written to this effect in the Journal Général de Médecine.

"If we examine the English authors on this subject, we shall find but a very small number in favour of contagion. Those English practitioners, however, who have resided longest in the West Indies, and who have had the widest field for observation in yellow fever, are not those who have written most on the disease. M. Valentin has conversed, or been in correspondence, with many of these, and they are entirely of his opinion. We must not, he remarks, be led away by Ling or Chisholm, whose minds were evidently biassed. ('Qui ont imprimé aux esprits une mauvaise direction.') The former had but a limited experience. He has given, indeed, a very good description of the epidemic which he witnessed at Charlestown; but he has mistaken the cause, which was local. He, as well as Rush, has led many practitioners into error. Besides, it is more than half a century since he saw the disease which he considered as imported; but Moultrie and Mackitrick were of a very different opinion. Chisholm only saw the yellow fever in 1792, 'et comme un Medecin Navigateur, s'est trop pressa d'ecrire: il s'est trompé dans presque tout ce qu'il avance.' Dr. Caldwell, of Philadelphia, has, in the Medical and Physical Memoirs, so refuted Dr. Chisholm, that the latter has been unable to reply. Rush, Moseley, and Miller, have also shewn the sandy foundation of Dr. Chisholm's doctrine.

"During three years that M. L Valentin resided in St. Domingo, only a few sporadic cases of yellow fever appeared in the hospitals of Cape François. But in other quarters it was prevalent, especially cially cially among the troops that were in the habit of bivouacing. At Jean Rabel, almost the whole of the soldiers were cut off by this scourge, towards the close of 1791-2; and this period, says he, certainly appeared the commencement of a new era in the icterode fever, which afterwards pressed so heavily on different parts of the colony, during the horrors of the revolution, and particularly after our flight to North America. M. Valentin is sure that those who came from Jean Rabel to the Cape were unaffected by fever, and were colonists, or those long resident in the climate. It was morally impossible, therefore, that the fever could be carried by any person from Cape François to Philadelphia in 1793, when the disease manifested itself epidemically in that ill-fated city. The Anglo-Americans, it is true, accused the new comers of having brought with them the contagion, although it was notorious, that no epidemic of the kind prevailed at St. Domingo at this period. The greater part of the refugees were landed tumultuously from the fleet of Vice-Admiral Cambi, at Norfolk, and other parts of Virginia, without incurring any such charge. Here they became domiciliated, and here M. Valentin established an hospital of the sick soldiers and sailors; yet not an inhabitant, native or stranger, exhibited any symptoms of yellow fever this year! All the physicians who have been sent out to St. Domingo, and who have long and carefully observed the disease in question, agree in denying contagion; and are satisfied, that yellow fever is endemic, and depending on local causes.

"M. Valentin has convinced himself, from extensive experience, and multiplied examples, that persons affected with yellow fever, when removed from the range of febrific causes to a more elevated and healthy situation, have never communicated the disease to others;—that individuals who have nursed the sick, in these latter places, and carried their clothes, or slept in their beds, before and after death, have not contracted the fever. Can as much be said of typhus?

"Those who have opened the bodies of yellow fever subjects, have always escaped the disease, when out of the range of the effluvia that produce it. M. Valentin has seen yellow fever patients carried to the hospitals, where no fever prevailed, and there die amid numbers of people affected with common complaints, without, in any instance, communicating it to the nurses or attendants. Inoculation with the saliva, serum, or matter ejected by vomit from the stomachs of yellow-fever patients—nay, the swallowing of these, have failed to propagate the disease.

"But let the inhabitants of a healthy spot remove to one where the yellow fever prevails, and consequently where the atmosphere is vitiated—let them pass a night, or even a few hours there, without entering a house where there is any sickness, and they will carry back with them, according to the degree of predisposition, the elements, the miasms, or, if you please, the germs of the disease, which will break out in the most salubrious situation a few

 days days afterwards, but without the power of extension among those who may nurse them.

"Of the numerous examples which might be adduced of yellow fever originating on board ships, M. Valentin brings forward the following:—The American ship Columbia, sailed from Providence during a season of health, and arrived at Marseilles in the month of August 1802, where she performed quarantine, having previously touched at two Spanish ports. During the voyage and quarantine, the crew had been perfectly healthy; but, on their being released from quarantine, the second and third mate, four seamen, and a negro, were successively attacked with yellow fever, and all died; three of them in three separate houses of the town, the others in the lazaret. Neither the nurses that attended, nor the surgeons that opened them, became affected with the disease; nor did any inhabitant of the town suffer from yellow fever. It was the same in 1804, when many of the Spanish, Danish, and Swedish, sailors died of yellow fever, without communicating the disease to any of their attendants or physicians. In these cases, a ship may be compared to a place on shore, where the air is stagnant, moist, warm, and tainted with various putrid effluvia. Of this one more remarkable instance may be related. A French flotilla, carrying troops from Italy to St. Domingo, sailed from Tarentum on the 2d of May, 1802; but, being buffeted by a storm in the Mediterranean, they put into several ports, particularly Leghorn, from whence they soon proceeded on their voyage. The provisions were bad, the salt-fish was rotten, and exhaled such an abominable odour, that it was thrown overboard. They were forced to put into Carthagena, where eight fresh transports were hired, on board of which, the troops and provisions were embarked. The San Nicolus de Varrios was appointed an hospital ship, and Dr. Beguerie was placed on board in charge of the sick. The three succeeding months of June, July, and August, were excessively hot. The yellow fever broke out early in the passage, and increased in violence as the ships approached the tropic. Dr. Beguerie having himself experienced an attack of yellow fever at Madagascar, had an opportunity, on landing at St. Domingo, of seeing the same disease there. He asserts, that there was scarcely a shade of difference between the yellow fevers of the east and west, and that with which the troops were harrassed on their passage from Europe.

"The history of the epidemic at New York and Philadelphia, in 1805, furnished abundant proofs of the truth of this position. About the middle of September, the atmosphere of New York was so embued with the cause of yellow fever, that in the course of two or three days after the first alarm was given, nearly fifty thousand people deserted the city, and fled in all directions. More than ten thousand persons established a kind of camp, or temporary town, on the elevated grounds of Greenwich; to which place they removed their merchandize, furniture, public offices, &c. &c. Those who were ill with yellow fever, accompanied the others, and many who who subsequently fell ill, afterwards joined them; yet, in no instance was the fever communicated or spread in the new residences. It was precisely the same at Leghorn, in 1804. Not one of the refugees who fled into the country and to Pisa communicated the disease to those among whom they resided.

"Such are the arguments and facts, by which M. Valentin attempts to demonstrate the non-contagious nature of yellow fever, and which we have not exaggerated or diminished. We think it our duty here, to notice a document in the New Medical and Physical Journal for July 1815, written by Mr. Amiel, surgeon to the forces of his Britannic Majesty at Gibraltar. In this, numerous facts, analogous to those brought forward by M. Valentin, appear equally to prove, that yellow fever is destitute of a contagious property. One of the most conclusive facts related by Mr. Amiel is this, that while the epidemic raged at Gibraltar, in 1814, the sick that were removed but a very short distance out of the town, in no instance communicated the fever to any one.

"A delicate task yet remains for us to accomplish.—Is the yellow fever contagious or not? In the course of our researches for the materials of this article, and in our conversations with physicians and other intelligent people, who have frequently witnessed yellow fever, we have ascertained, that the general opinion among the most able and distinguished judges is, that the yellow fever is endemic in the greater number of places where it appears; that some epidemics are contagious, while others are not so; and, in fine, that the disease may be sometimes imported; and is capable of developing itself sporadically in those places 'qui reunissent les conditions de l'endemic.'"

Often as we have regretted the loose language of our countrymen, we expected something more precise from a national work, (the French Dictionary of Medical Knowledge,) and in a language which boasts of peculiar purity and correctness. Let us see how far these are exemplified in the above paper. The first inquiry is very properly into the meaning of the word *contagion*. In this, a sort of reference is made to Dr. Hosack's paper, but the question is left, if possible, more undecided than by the American physician; and even the terms *endemic* and *epidemic* are used as synonymous, or, at least, without any distinction that can be collected from the writers.

The greater part of the paper contains the contradictory narrations of, we have not taken the trouble to count how many, different writers, and their various opinions concerning the contagion of yellow fever. Instead of this, we expected their facts, the credit due to each, and the *inference* of the compilers; not the opinions of the writers, any further than as it may have influenced their historical statements. Lastly, we have the accomplishment of the *delicate task* imposed on the compilers—"Is the yellow fever contagious or not?" and here, as Johnson concludes his Rasselas, we have a "conclusion in which nothing is concluded." We are glad, however, however, to find the paper intelligible to some of our countrymen, but cannot help expressing a wish to know what their opinions are, "if so nearly similar to the above." All this absurdity might be passed over with a smile, were it not for a malicious grin at a most respectable character, and conveyed to a whole nation.

"If this be true," says the writer, "we can attach little importance to the testimony of that physician who could thus prostitute his talents and influence at the shrine of interest. This is another specimen of American veracity."

We had the happiness of Dr. Rush's correspondence whilst living, and lament, in common with every friend to science and humanity, his early death; for, of such a man, we should for ever say, *serus in cœlum redeat.*

We shall take this opportunity of introducing part of the last printed work of the late Dr. Lettsom, which he calls Recollections of Dr. Rush, and which, we believe, was never published, but presented to his friends.

"Painful must it be to every man of science, and particularly to every man of medical science, to reflect, that he, who for the space of nearly half a century, had been the means, by his discoveries and judgment, of respiting thousands from the grave, is now himself an inhabitant of that final receptacle of mortality. I allude to Dr. Rush, the Sydenham of America, who died in Philadelphia, on the 19th of April, 1812. The letter which announced this event gives me the following information:—

"'We have been afflicted with an epidemic, termed Typhus, or Spotted Fever (which has prevailed for some years in the New-England States), for the last two months. It, being in general under the control of medicine, has not proved very fatal. The symptoms are, a general prostration of strength. The remedies which have proved most successful have been stimulants—large quantities of brandy taken inwardly, &c. In his own case, Dr. Rush mistook it for the pleurisy, and was freely bled, which is thought to have occasioned his death.'

"I have no account of his age, but conclude, from circumstances, that he had attained about his 69th year. He was educated in Philadelphia, and completed his medical studies in Edinburgh, at the university of which he received his degree of doctor of medicine, in the year 1768, after presenting and defending his Thesis, *De Coctione Ciborum*, a performance so accurate in experiment, and so ingenious and lucid in diction, as to have placed him in a prominent and honourable point of estimation in that celebrated school, which a Munro raised, and a Cullen supported and dignified.

"On his return from Edinburgh, I first met him at the house of that celebrated physician, Dr. John Fothergill, with whom I then resided; and, from that period to the time of his decease, our correspondence was maintained by an uninterrupted intercourse of literary communication: the first letter I received being dated from Philadelphia,

Philadelphia, November 29, 1771, soon after his return to that city."

After mentioning several productions, some of which are not connected with medicine, Dr. L. continues, "I have enumerated singly these performances of the professor, as they are less known than his great work on the yellow fever, which, with his publication on the remittent bilious fever, and numerous other interesting and luminous essays, are collected into six octavo volumes,* and now enrich the libraries of many medical practitioners in Europe. The improvements they have produced in the treatment of disease being generally known and practised, it is less requisite to dwell upon their merits; but I cannot totally omit noticing the vast effort of genius and science; the novelty and bold decision of medical practice; and the amplitude of experiment, which his great work on the yellow fever every where elicits.

"When this grand production, uniting, in an almost unprecedented degree, sagacity and judgment, first appeared, Europe was astonished. Even at this moment, I cannot recal to mind the phenomena connected with the rise, progress, and effect, of that dreadful malady, without admiration; and the conduct of the physician without veneration. Contemplate this illustrious professor, emerging from the prostration of strength induced by this fever; his aged mother dying; his sister a corpse; his pupils dead around him; flying from house to house, wherever infection is raging; at home, his apartments filled with supplicants diseased and dying; Death almost every where stalking over the victims from the raging pestilence. If there is an example of heroic fortitude—of disinterested exposure to peril for the public welfare any where recorded in medical history, superior to those which dignified this unappalled and luminous philanthropist, let that character be venerated as one of the first benefactors of mankind. He says, indeed, of himself, 'It was meat and drink to me to fulfil the duties I owed to my fellow citizens in this time of great and universal distress.' He adds, 'Between the 8th and the 15th of September, I visited and prescribed for between one hundred and one hundred and twenty patients a day. Several of my pupils visited a fourth or fifth part of that number. For awhile we refused no calls. In the short intervals of business which I spent at my meals, my house was filled with patients, chiefly the poor, waiting for advice. For many weeks I seldom ate without prescribing for numbers as I sat at my table.'

"By incessant employment, day and night, he unavoidably became greatly indisposed; but let Humanity mark the disregard of himself, and Sympathy pause upon his anxieties and sufferings. He observes, 'From all these sources, streams of contagion were constantly pouring into my house, and conveyed into my body by the air, and in my aliment. Thus charged with the fuel of death, I

"* The first published in 1789, the last in 1811."

was was frequently disposed to say, with Job, and almost without a figure, to 'corruption, thou art my father; and to the worm, thou art my mother and my sister.' The next day (recovering from illness) I came down stairs, and prescribed in my parlour for not less than one hundred people. On the 19th of the same month I resumed my labours, but in great weakness. It was with difficulty that I ascended a pair of stairs by the help of a banister.'

"When I communicated an account of this mortal disease, and of the conduct of some of the medical professors in Philadelphia who deserted the city at this alarming crisis, to my admired correspondent, the celebrated Zimmerman, whose recent death every lover of virtue and science must deeply lament, he favoured me with the following reflections:

"'La conduite, moralement très pétite du Dr. ****, pour lequel les habitans de Philadelphie avoient le plus de confiance, resembloit parfaitement à celle du grand Sydenham du temps de la Peste de Londres. La conduite du Dr. Rush a merité, que non seulement la ville de Philadelphie, mais l'humanité entière lui elève une Statue." This letter, which is very long and very interesting, is dated Hanover, May 27, 1794.

"With Zimmerman, I presume every friend of liberal science will conclude, that it would be an act of gratitude in his contemporaries, and of honour in his countrymen, to erect such a memorial to his memory.

"Were I to determine the station of our deceased friend, I should place him with Hippocrates and Sydenham, and individually designate him the Sydenham of America.

"In the inestimable work of Sydenham, we contemplate minuteness of perception and depth of meditation, constituting a mental energy which enabled him to discriminate facts, and, with a vivid penetration, to form a beautiful concatenation of experience that perhaps was never equalled in medical science. Rush approached, if not exceeded, him in grandeur and compass of thought, though less discriminating in that felicitous arrangement of medical phenomena which distinguish Sydenham, whilst his theories were less consonant to nature. Rush possessed a lively imagination, which his extent of judgment could alone control, and lead to important discoveries; and though he sought to rise above the trammels of established opinions, and delighted to grasp at novelties, he did not wish to be paradoxical. If he acquired less felicity of method and arrangement than Sydenham, he rivalled him in vigour of investigation, and still more in boldness and decision of professional practice. To Sydenham, the motto of '*conamen naturæ*' is most applicable.—To Rush, I would appropriate that of '*nullius in verba.*' Time will more fully appreciate their relative abilities; and the next century will place them as coadjutors in the grand development of medical knowledge."

Who the author of the above account of Dr. Rush's death was, Dr. Lettsom does not inform us, nor are we certain that the intelligence telligence was not collected from an anonymous paper in a periodical publication. It is, however, probable, that the supposed mistake of the deceased as to his own case gave rise to this absurd story of his recantation, and the still more wicked story of his living so many years with a constant lie in his mouth, to answer a particular purpose.

The cold calumnious suggestion is less remarkable in a Frenchman, who may have been unacquainted with Dr. Rush's character, and, from his imperfect knowledge of his language, may have fallen into an error by a misconception of the report. But we cannot make the same excuse for an English writer; nor does his qualifying IF, in our opinion, at all mend his case.

It would at least be decent, not to say candid, to wait for better information, when the friends of the deceased may so far have recovered from their loss as to favour the world with some authentic memoirs. Authentic accounts of his death have, indeed, arrived, which contradict even the supposed error concerning his own case. This will be found in our account of "Transactions of Societies," at the close of this Number.

Having hinted that the above report from the French dictionary is, in many respects, similar to Dr. Hosack's, we shall transcribe from that writer the passage in which he enters into the discrimination of various kinds of contagion. It is contained in an appendix to his paper, which we have for some time promised to notice.

"The communication of this virus from the sick to the well, in whatever form it may be conveyed, as uniformly produces the *same disease*, as *inoculation* excites the *small-pox*, or *vaccination* conveys the *vaccine virus*. So far, then, there is something in *common* in the communication of contagious or infectious diseases, which should be accordingly expressed in the language we employ—some of those diseases are conveyed in one form, others in a different; we should then be equally careful to mark those circumstances in which they *differ*, as well as those which they possess in *common*.

"Such an arrangement appears to me not only practicable, but, at the same time, calculated, in some degree, to harmonize the differences of opinion which now separate the contagionists and non-contagionists. Under these impressions, I propose to arrange those diseases which are communicable from one to another under three heads. First, those which are communicated *exclusively* by *contact*. In this class I enumerate the itch; syphilis; the sibbens of Scotland; the laanda of Africa; frambæsia, or yaws; elephantiasis, or leprosy; hydrophobia; and the vaccine virus.

"Neither of those diseases can be communicated in any other way than by *contact;* they are, therefore, *contagious* diseases, in the strict etymological sense of the term. It is also to be remarked, that these diseases are never conveyed through the medium of the *atmosphere;* actual contact alone can communicate them from one person to another.

"These diseases, acknowledged by all to be contagious, and so denominated by all writers, have a law of communication peculiar to themselves. But there is a second class of diseases, also considered as contagious, which are communicated under different circumstances, governed, in this respect, by different laws of communication.

"Those to which I now allude are such as are communicated both by *contact* and the *atmosphere.* In this class I arrange small-pox, measles, chicken-pox, hooping-cough, scarlet fever, and cynanche maligna.

"Contact, or the *close approach* to the sick, labouring under these diseases, will communicate them to those who are susceptible of their influence—but they are no less communicable through the *medium of the atmosphere.* A *second law,* which governs the communication of this class of contagious diseases, is, that they are communicable in *every season,* in the heat of *summer,* as well as in the cold of *winter*—in a *pure* as well as in an *impure* air, though more readily by the latter than the former. A *third law* of communication in this class of diseases is, that the persons afflicted with them are not generally susceptible of a second attack. I say *generally,* because exceptions are related upon very respectable authority.

"This second class of contagious diseases is, therefore, abundantly distinguished from the first; but they are still associated by most medical writers under the same head of contagious diseases, without assigning to each class its discriminating character.

"The same want of discrimination has, in my opinion, occasioned the numerous disputes among physicians relative to the contagiousness and non-contagiousness of those fevers which I enumerate as the *third* class of diseases that are communicable from one person to another. Under this head I arrange plague; yellow fever; typhus, *jail, ship, hospital,* or *lake fever;* and dysentery.

"These diseases are only, in general, communicable through the medium of an *impure* atmosphere: in a pure air, in large and well-ventilated apartments, when the dress of the patient is frequently changed, all excrementitious discharges immediately removed, and attention paid to cleanliness in general, these diseases are not communicated, or very rarely so, from one to another. But in an *impure* air, rendered so by the decomposition of animal and vegetable substances, as takes place in low marshy countries, or by concentrated human effluvia, as in camps, jails, hospitals, or on shipboard, they are rendered not only extremely malignant and mortal in themselves, but become communicable to others who approach the sick, or breathe the same atmosphere, which has become *assimilated* to the poison introduced, insomuch that *the*

*same*

*same specific disease* is communicated, whether it be the *plague, yellow fever, typhus,* or *dysentery.*"

At present we shall only remark that the French report has one advantage over the above—*Contagium a contactu.* Now, what contact with a sick person, or with the effluvia from him, is necessary to induce yellow fever or dysentery? Do they not arise from a constitution of the atmosphere, and spread by a similar cause? The Frenchmen, therefore, are more prudent than Dr. Hosack; they keep clear of the word infection, not knowing how to manage, and well aware that synonyms in philosophy are never admitted. Their etymology of contagion is unexceptionable. Infection, from *inficere,* to die or taint, admits of a more enlarged meaning, in which contact with the disease itself is unnecessary.

What is here offered is only to invite the communications of our correspondents, before we enter more systematically on these subjects.

